# Comparative venomics of *Psyttalia lounsburyi* and *P. concolor*, two olive fruit fly parasitoids: a hypothetical role for a GH1 β-glucosidase

**DOI:** 10.1038/srep35873

**Published:** 2016-10-25

**Authors:** Hugo Mathé-Hubert, Dominique Colinet, Emeline Deleury, Maya Belghazi, Marc Ravallec, Julie Poulain, Carole Dossat, Marylène Poirié, Jean-Luc Gatti

**Affiliations:** 1 Université Côte d’Azur, INRA, CNRS, ISA, France; 2 CNRS, Aix-Marseille Université, UMR 7286, CRN2M, Centre d’Analyses Protéomiques de Marseille (CAPM), Faculté de Médecine, Marseille, France; 3 INRA, Univ. Montpellier, UMR 1333 « Microorganism & Insect Diversity, Genomes & Interactions » (DGIMI), CC101, Montpellier Cedex 34095, France; 4 Commissariat à l’Energie Atomique (CEA), Institut de Génomique (IG), Génoscope, 91000, Evry, France

## Abstract

Venom composition of parasitoid wasps attracts increasing interest – notably molecules ensuring parasitism success on arthropod pests – but its variation within and among taxa is not yet understood. We have identified here the main venom proteins of two braconid wasps, *Psyttalia lounsburyi* (two strains from South Africa and Kenya) and *P. concolor,* olive fruit fly parasitoids that differ in host range. Among the shared abundant proteins, we found a GH1 β-glucosidase and a family of leucine-rich repeat (LRR) proteins. Olive is extremely rich in glycoside compounds that are hydrolyzed by β-glucosidases into defensive toxic products in response to phytophagous insect attacks. Assuming that *Psyttalia* host larvae sequester ingested glycosides, the injected venom GH1 β-glucosidase could induce the release of toxic compounds, thus participating in parasitism success by weakening the host. Venom LRR proteins are similar to truncated Toll-like receptors and may possibly scavenge the host immunity. The abundance of one of these LRR proteins in the venom of only one of the two *P. lounsburyi* strains evidences intraspecific variation in venom composition. Altogether, venom intra- and inter-specific variation in *Psyttalia* spp. were much lower than previously reported in the *Leptopilina* genus (Figitidae), suggesting it might depend upon the parasitoid taxa.

Hymenopteran parasitoids represent 10 to 20% of all insect species, being as such one of the largest group of venomous organisms. They develop on (ectoparasitoids) or inside (endoparasitoids) other arthropods, consuming their tissues and ultimately killing the host. They are thus important regulators of arthropod populations in nature, and used as pest control auxiliaries in agriculture^1^. One of the challenges faced by endoparasitoids is overcoming the immune response of the host, i.e. the formation of a multicellular, melanized capsule around the parasitoid egg^2^. To ensure successful parasitism, endoparasitoids have thus evolved original strategies, the most common being the injection with the egg of various components that manipulate the host physiology (i.e. immunity, metabolism, reproduction, molting) and behaviour (i.e. movements, feeding). These components are often a complex mixture of ovarian and venom proteins^3^,^4^, but they also include virus-like particles (VLPs)^5^ or wasp-specific polydnaviruses (PDVs)^6^.

Broad studies reporting transcriptomic and/or proteomic analyses have recently improved our knowledge of venom composition in different parasitoid families, evidencing its high complexity and diversity^3^,^4^. Indeed, although some venom proteins – e.g. serine proteases, metalloproteases or esterases – are largely shared by parasitoids, others seem specific to a parasitoid group or even species, and some are only found in a few phylogenetically distant species^3^. This suggests a rapid evolution of parasitoid venom composition, based on unidentified molecular mechanisms.

Strikingly, a high venom diversity was observed between the closely related *Drosophila* parasitoids *Leptopilina boulardi* and *L. heterotoma* (Hymenoptera, Figitidae), with none of the abundant venom proteins in common^7^. To assess whether this variation between two figitid species that differ in their host range similarly exists in other parasitoid taxa, we compared here the venom composition of two braconid wasps, *Psyttalia lounsburyi* and *P. concolor* (Hymenoptera, Braconidae, Opiinae) that belong to the same complex of species^8^. Both *Psyttalia* species are used as biological control agents of the olive fruit fly *Bactrocera oleae*^9^ and they differ in their host range. *P. lounsburyi* is specialized on *B. oleae*^9^ whereas *P. concolor* successfully develops in *B. oleae* and at least 13 other fruit fly species^10^. Comparison of *P. lounsburyi* and *P. concolor* venom was performed using a combined transcriptomic and proteomic approach, and it was extended at the intraspecific level using two geographically distant African strains of *P. lounsburyi* (South Africa and Kenya). We also compared data with large-scale venomics results from other braconids, either associated with PDVs, as *Chelonus inanitus*^11^ and *Microplitis demolitor*^12^, or devoid of PDVs, as *Aphidius ervi*^13^, since using various parasitism strategies could possibly impact venom evolution and composition.

This study will contribute to a better picture of the diversification of venom components at a short evolutionary scale, opening the way to the characterization of underlying mechanisms.

## Results and Discussion

### Structure of the *Psyttalia* venom apparatus

**Figure 1:**
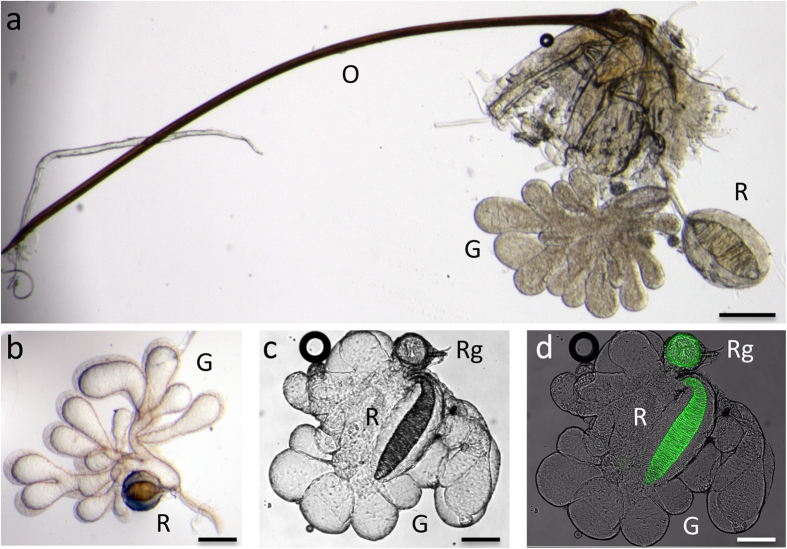
Microscopy observation of the venom apparatus of *Psyttalia* species. *Light microscopy*: (**a**) *P. lounsburyi* female venom apparatus composed of a multi-lobed gland (G), a reservoir (R) and a long ovipositor (O); (**b**) Dissected *P. lounsburyi* venom gland showing the thick tissue envelope of the gland and the basal lateral branching of the reservoir; (**c**) *P. concolor* venom apparatus evidencing the small round gland at the base of the apparatus (Rg); (**d**) the same, overlaid with a fluorescence micrograph showing the green auto-fluorescence of the internal spirals of the reservoir and the small round gland. Bars = 100 μm.

As typically observed for Braconidae, the venom apparatus of both *Psyttalia* species is composed of venom gland filaments (which secrete venom), a venom reservoir, and a venom duct that extends into the ovipositor (Fig. 1a). As was previously described in Opiinae^14^,^15^, *P. lounsburyi* and *P. concolor* venom gland is multi-lobed, each lobe displaying an external thick layer of tissue and a central lumen filled by a large volume of venom (Fig. 1b,c). The gland lobes are joined together at the base where the ovoid shaped reservoir is connected. The reservoir is composed of a large muscular layer surrounding a small internal volume of venom, suggesting it may serve as a “pump” for injecting venom at the time of oviposition rather than as a storage organ. The reservoir also shows internal structures that form intricate spirals possibly involved in maintaining the shape of the reservoir, like spiral springs, by passively counteracting the muscular contraction (Fig. 1b,c). At the base of the venom apparatus of *P. concolor*, we also observed a “round gland” filled with large vesicles (Fig. 1c), already described by Quicke^14^ and called “basal bulb” by Wharton^15^. Interestingly, the round gland and the intima spirals of the reservoir showed a strong endogenous green fluorescence (Fig. 1d), suggesting the presence of universal cellular auto-fluorophores such as NAD(P)H and flavins, pigments, or cuticular compounds^16^.

### No VLP or PDV in *P. lounsburyi* and *P. concolor*

**Figure 2:**
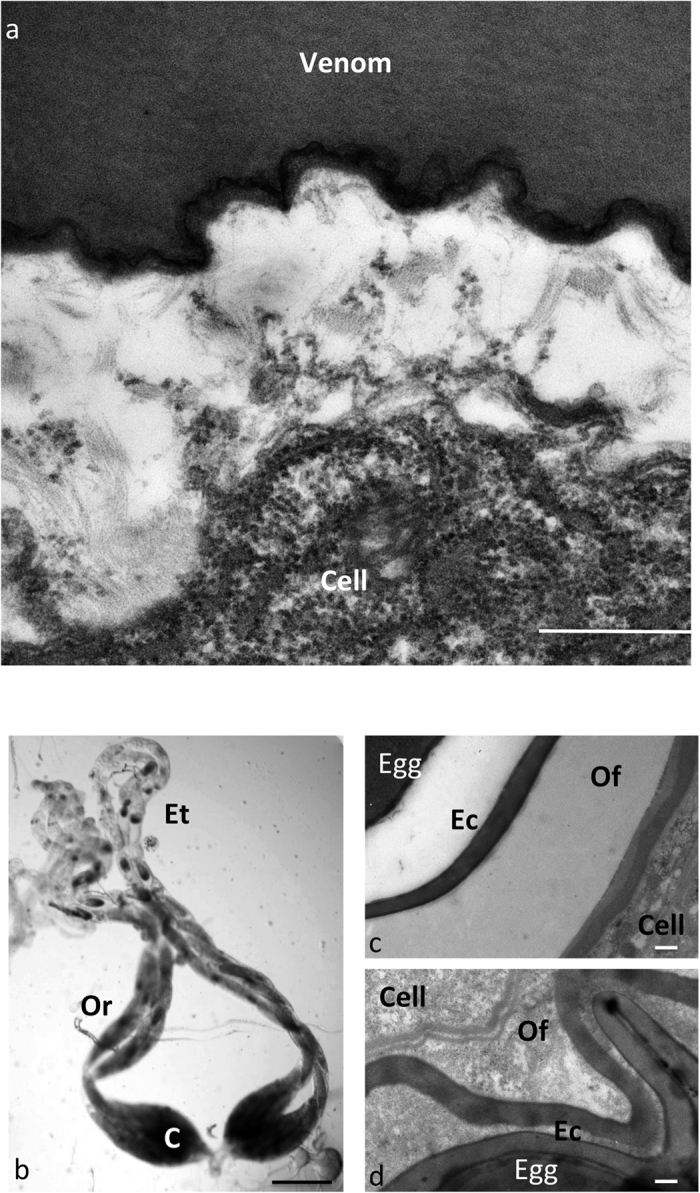
Microscopy observation of venom glands and ovaries from *Psyttalia* species. (**a**) Electron microscopy picture of a transversal section through a venom gland lobe of *P. concolor*, showing the rough endoplasmic reticulum-rich cytoplasm of the cell and the venom fluid. The empty space is due to retraction during dehydration. No vesicle is observed in the size range of the VLPs described in parasitoids venom (50 to 100 nm). Bar = 500 nm. (**b**) Picture of whole mounts *P. concolor* ovaries. Oogenesis occurs in egg tubes (Et), late oocytes being located in reservoirs (Or) and moving down to the calyx (C) where tubes fuse to form the oviduct. Bar = 500 μm. (**c,d**) TEM micrographs of sections through the calyx region of *P. lounsbury* (**c**) and *P. concolor* (**d**) ovaries, showing the egg chorion (Ec), the ovarian fluid (Of) and the calyx cells. No PDV particle is observed inside the cells nor in the fluid surrounding the egg chorion. Bar = 500 nm.

Among the few studies on *Psyttalia* species, two had reported the occurrence of unidentified virus-like particles (VLPs) within the venom secretory cells (in *P. concolor*, previously *Opius concolor*^17^, and the close species *O. caricivorae* Fisher^18^). We thus performed electron microscopy on *P. concolor* venom glands to search for VLPs, as well as on *P. lounsburyi* and *P. concolor* ovaries to ensure the absence of PDVs (polydnaviruses). We did not observe VLPs or vesicular material resembling VLPs in venom gland cells or venom (Fig. 2a), nor viral structures or PDV particles in the ovarian cells and fluid close to the eggs (Fig. 2b–d). Accordingly, Pl and Pc transcriptomes contained no transcript having similarities with genes specific of nudiviruses (from which braconid PDVs derive) or the sister group of baculoviruses. As coronaviruses and cypo-like viruses were also described in *P. concolor* venom glands^17^, previous observations likely corresponded to small viruses infecting the reproductive tract of the samples, as reported also in other Hymenoptera^19^. In the absence of VLPs and PDVs, secreted venom proteins are likely the main maternal actors of parasitism success of *Psyttalia* species.

### Comparison of electrophoretic profiles of *Psyttalia* venom proteins

**Figure 3:**
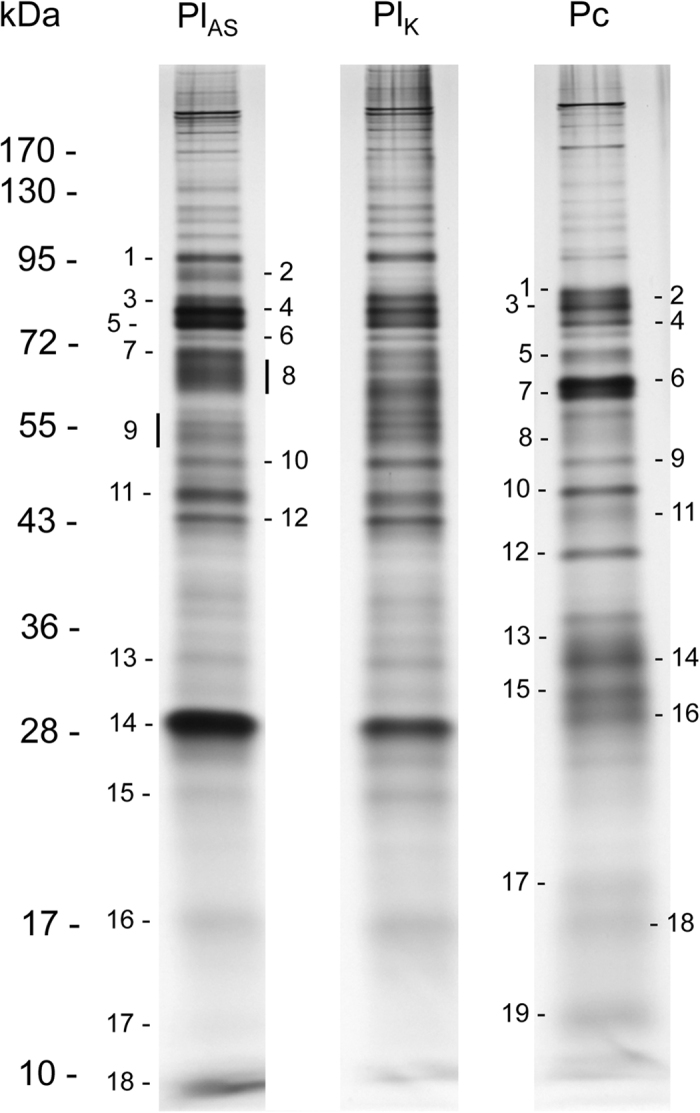
1D SDS-PAGE separation of *P. lounsbury* and *P. concolor* venom proteins. Venom proteins from 50 Pl_SA_, Pl_K_ and Pc females were separated on a 6–16% SDS-PAGE under reducing conditions and visualized by silver staining. All stained protein bands numbered on the gel were excised and submitted for protein identification by LC-MS-MS. Molecular weight standard positions are indicated on the left (kDa).

**Figure 4:**
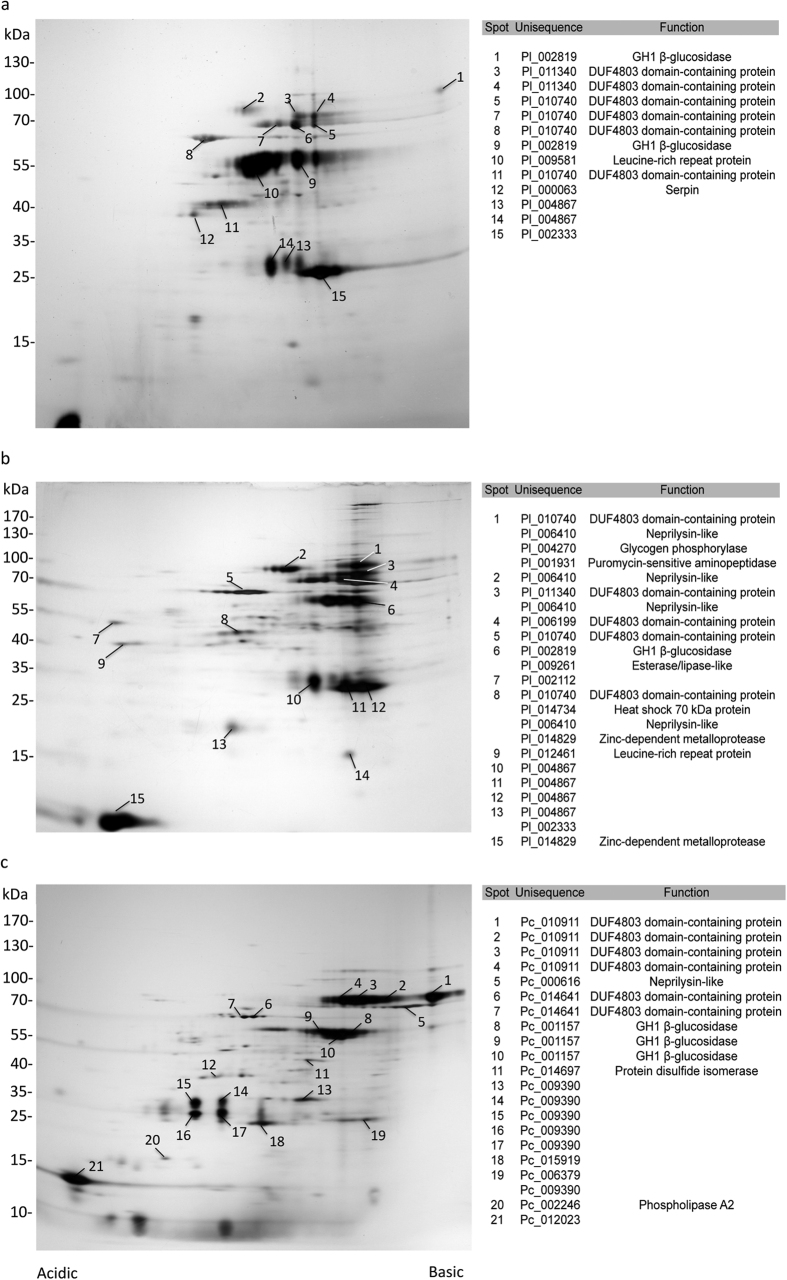
2D SDS-PAGE separation of *P. lounsbury* and *P. concolor* venom proteins. Venom proteins from 50 Pl_SA_ (**a**), Pl_K_ (**b**) and Pc (**c**) females were separated by IEF followed by 6–16% SDS-PAGE. Following silver staining, the major spots (numbered) were cut and analyzed by LC-MS-MS. Spots for which a protein with a putative function was identified are indicated on the right. Molecular weight standard positions are indicated on the left (kDa).

Venom samples collected from 50 individuals of *P. concolor* (Pc) and the *P. lounsburyi* South African (Pl_SA_) and Kenyan (Pl_K_) strains were analyzed by 1D and 2D gel electrophoresis (Figs 3 and 4). On a 6–16% SDS-PAGE, the protein content of venom glands was resolved into numerous bands from 10 kDa to more than 200 kDa (Fig. 3). On the silver-stained 2D gels, 50 to 100 spots were clearly visible, having a 4 to 8.5–9 isoelectric point, and ranging from 10 to more than 120 kDa (Fig. 4). Some trains of spots were also observed that likely corresponded to post-translational modifications of the same protein.

The Pl_SA_ and Pl_K_ 1D electrophoretic profiles were very similar with presence/absence or intensity variation detected for only a few bands (Fig. 3). The distribution of 2D spots evidenced more differences, in particular the absence of the Pl_SA_ spot number 10 (55 kDa) in the Pl_K_ strain (Fig. 4a,b). We observed a greater variation at the interspecific level with several profile differences in the 25–35 kDa range (Figs 3 and 4).

### Comparison of transcriptomic and proteomics data between *Psyttalia* wasps

All the major bands and spots on 1D and 2D electrophoretic profiles of Pl and Pc venom, and a number of minor spots (70 bands for Pl, 46 bands for Pc, from at least two 1D gels per species; 117 spots for Pl, 57 for Pc, from at least three 2D gels per species) were excised, and tryptic peptides were analyzed by LC-MS-MS. In parallel, a transcriptomic analysis of Pl and Pc venom glands was performed, based on Illumina sequencing. *De novo* assembly of the Pl transcriptome was improved using additional 454 (full body) and Sanger (venom apparatus) sequence data (Supplementary Figure S1) and it yielded a total of 16,943 and 16,360 unisequences for Pl and Pc, respectively (Supplementary Table S1). Data suggested a similar quality of the transcriptomes, based on general features and similarity searches (Supplementary Table S1), as well as GO terms comparison (Supplementary Figure S2).

The combined transcriptomic and proteomic data resulted in 39 and 40 unisequences for Pl and Pc, respectively, among which 32 and 36 had a putative function (Supplementary Tables S2 and S3). Some of these, such as actin-5C or glyceraldehyde-3-phosphate dehydrogenase 2, were typical cellular proteins with no predicted signal peptide (Supplementary Tables S2 and S3). Whether their identification in venom is due to cellular damage during collection, or these proteins are actually secreted by non-canonical export mechanisms remains unclear. Therefore, we only considered as putative venom proteins the unisequences (i) found in venom proteomics, and (ii) either predicted to be secreted or for which the presence of a signal peptide was not tested due to the incompleteness of the sequence. This resulted in a total of 32 and 30 putative venom proteins for Pl and Pc respectively (Tables 1 and 2), whose relative abundance was compared using (i) the RPKM normalized number of Illumina reads from Pl and Pc venom apparatus, mapped to the assembled transcriptomes and (ii) the number of peptides matches in Mascot searches.

Interestingly, most of the proteins identified in the proteomics of the reservoir (detection of the most abundant putative venom proteins only, data not shown), such as actin or paramyosin, had a predicted muscular function, as expected from microscopy observations (see above; Fig. 1).

### Global analysis of *Psyttalia* wasps venom proteins and comparison with other wasps

**Figure 5:**
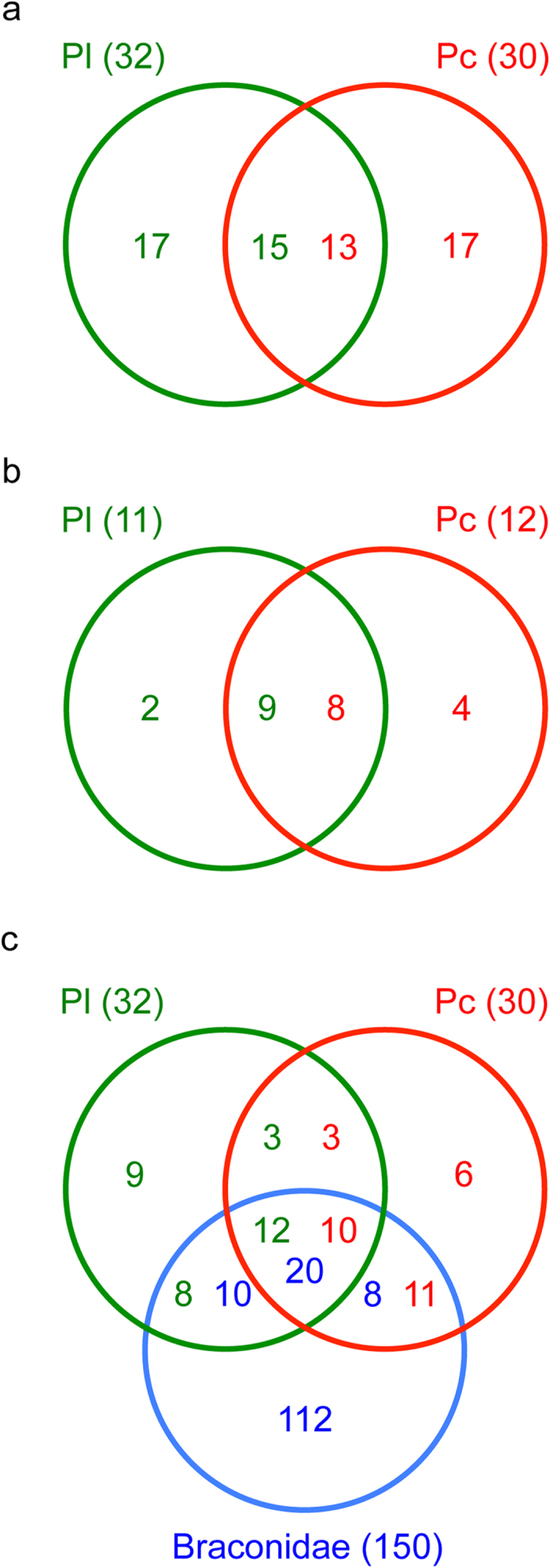
Venn diagrams showing the number of analyzed venom proteins shared between the following species. (**a**) *P. lounsburyi* and *P. concolor*; (**b**) *P. lounsburyi* and *P. concolor*, considering only the abundant proteins (RPKM > 50 and Mascot matches > 10); (**c**) *P. lounsburyi*, *P. concolor* and other Braconidae species.

Comparison of venomics data from the two *Psyttalia* species evidenced that 47% and 43% of the proteins identified in Pl and Pc were shared with the other species, respectively (Tables 1 and 2; Fig. 5a). In comparison, *L. boulardi* and *L. heterotoma* shared less than 20% of the identified venom proteins^7^. When considering only the most abundant venom proteins (RPKM > 50 and Mascot matches > 10), 9 and 8 out of the 11 Pl and 12 Pc proteins, respectively, were shared between *P. concolor* and *P. lounsburyi* (Fig. 5b; Tables 1 and 2) whereas the two *Leptopilina* species had no protein in common^7^. Finally, 20 Pl and 21 Pc venom proteins (63% and 70%) had already been identified in the venom of another braconid species (Fig. 5c; Tables 1 and 2).

### Identified venom proteins

Putative venom proteins described below are classified based on their abundance in venom (RPKM values and Mascot matches; Tables 1 and 2) and their occurrence in the venom of (i) both *Psyttalia* species, (ii) *P. lounsburyi* only and (iii) *P. concolor* only. Venom proteins with a putative function are described in Table 3 (with the proposed biochemical function, previous identification in parasitoid venom, and demonstrated or proposed role in parasitism success). Several proteins with low RPKM values and lacking N-terminal sequence were not considered since they were typical cellular proteins and the number of proteomic matches was low (Tables 1 and 2).

#### Proteins identified in the venom of both Psyttalia species

##### Proteins of unknown function

Several abundant unisequences with no predicted function were characterized by a high number of matches (Tables 1 and 2) and rather intense protein spots (Fig. 4). Among these, a family of five related proteins contains a signal peptide and the DUF4803 domain of unknown function (Supplementary Figure S3). They share similarities with venom proteins of the braconids *C. inanitus*^11^, *M. demolitor*^12^ and *M. hyperodae*^20^, and of *N. vitripennis*^21^.

##### Leucine-rich repeat protein

Two and four unisequences encoding leucine-rich repeat (LRR) proteins were identified in Pl and Pc, respectively (Tables 1 and 2; Supplementary Tables S2 and S3). One of these (Pl_009581) was highly abundant (rank 2) in the *P. lounsburyi* SA strain only, and it corresponded to one of the most intense spot (spot 10, Fig. 4A). The complete unisequences contain a N-terminal signal peptide and 9 to 19 canonical LRR motifs similar to the LRR motif in Toll-like receptors (TLRs). Yet, the majority of TLRs are multidomain proteins while *Psyttalia* predicted proteins only contain the LRR domain, as already observed for *A. ervi* venom LRR proteins^13^. As suggested for *A. ervi*, *Psyttalia* truncated LRR proteins might interfere with the host immune response by targeting the Toll pathway.

##### Neprilysin-like metalloproteases

Three and two unisequences encoded neprilysin-like (NEP) zinc-dependent metalloproteases in Pl and Pc, respectively (Tables 1 and 2; Supplementary Tables S2 and S3), one of which was in high abundance (rank 8; 37 and 47 peptide matches in Pl and Pc venom, respectively). NEP-like proteins occur in the venom of several parasitoid wasps (Table 3).

Another zinc-dependent metalloprotease was identified in each *Psyttalia* wasp, with low inter-species sequence similarity. Both proteins are weakly related to venom reprolysin-like proteins of *P. hypochondriaca*^22^ and *Eulophus pennicornis*^23^. However, the sequences were incomplete and the number of matches in the venom was rather low (Tables 1 and 2; Supplementary Tables S2 and S3).

##### GH1 β-glucosidases

Peptides from major 2D spots at 55–60 kDa (spots 6 and 9 in Pl_K_ and Pl_SA_, respectively, spots 8, 9, 10 in Pc) matched with two unisequences (one for each *Psyttalia* species) that encoded proteins containing a glycosyl hydrolase family 1 (GH1) domain (pfam00232) found in GH1 β-glucosidases (Fig. 4). The high RPKM values and number of peptides matches confirmed that the Pl_002819 and Pc_001157 unisequences, that share 97% identity (Supplementary Figure S4), were among the most abundant in venom (Tables 1 and 2; Supplementary Tables S2 and S3). The Pc sequence seems full-length, with a predicted signal peptide of 18 residues, while the Pl sequence probably lacks the N-terminal part (Supplementary Figure S4). The 56.5 kDa predicted MW of the mature protein is close to that of the observed spot on 2D gels, suggesting none or few glycosylation, although several N-glycosylation sites are predicted. Yet, several spots were observed, having different isoelectric points, suggesting post-translational modification(s) and thus several isoforms (Fig. 4). Two Pl and one Pc additional unisequences, not found in proteomics, shared similarities with GH1 β-glucosidases, suggesting occurrence of a multigene family (Supplementary Figure S5). GH1 β-glucosidases were previously identified in venomics data from *M. demolitor* and *A. ervi,* but they did not correspond to abundant proteins (Tables 1, 2 and 3). Finally, the venom of the ichneumonid *Pimpla hypochondriaca* was reported to display β-glucosidase enzymatic activity (Table 3).

GH1 β-glucosidases are found from bacteria to mammals. They play an essential role in the metabolism of glycolipids and exogenous glycosides by hydrolyzing glycosidic bonds and removing non-reducing terminal glucosyl residues from saccharides and glycosides. This enzyme family includes for instance the myrosinases, well-known for their role in the “glucosinolate-myrosinase” plant defense system (Table 3), and also identified in the cabbage aphid *Brevicoryne brassicae* that feed on crucifers^24^.

**Figure 6:**
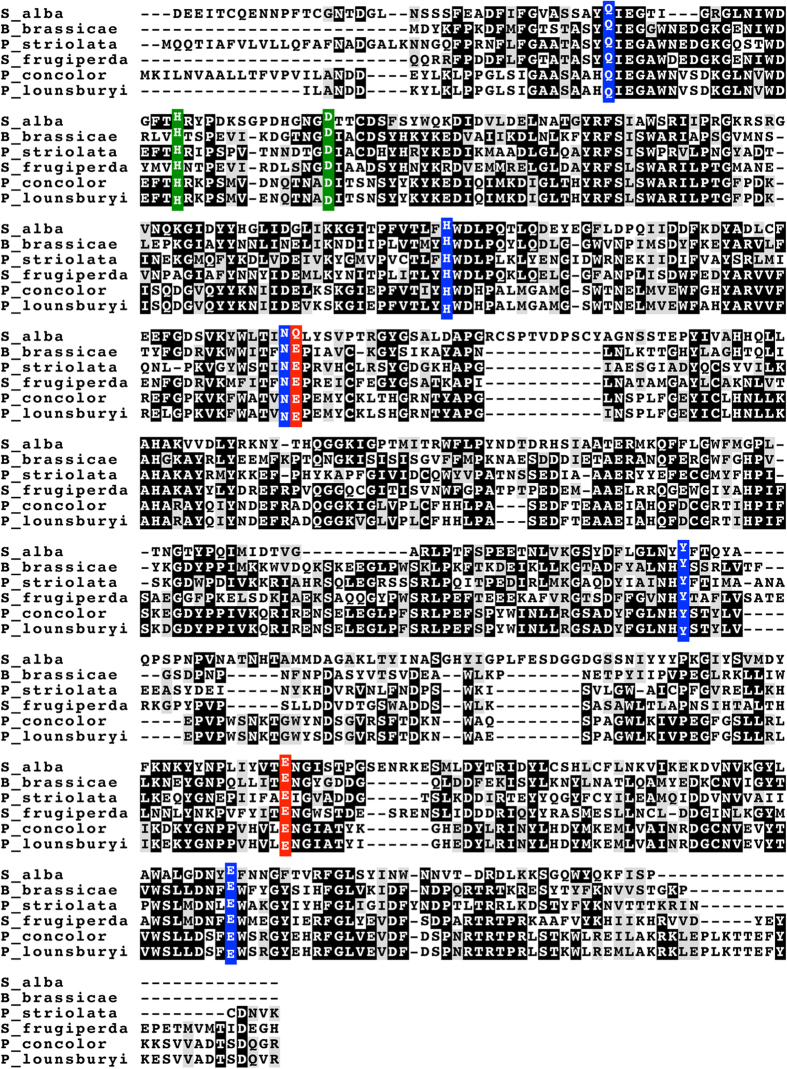
Multiple alignment of GH1 β-glucosidase sequences. Identical and similar residues are highlighted in black and grey, respectively. Catalytic residues are printed in white on a red background. Ligands of the Zn^2+^ ion are printed in white on a green background. Residues involved in glucose-ring recognition are printed in white on a blue background. S_alba, *S. alba* (1MYR_A); B_brassicae, *Brevicoryne brassicae* (1WCG_A); P_striolata, *Phyllotreta striolata* (AHZ59651); S_frugiperda, *Spodoptera frugiperda* (5CG0_A); P_concolor, *P. concolor (*Pc_001157); P_lounsburyi, *P. lounsburyi (*Pl_002819).

The alignment of Pl and Pc venom unisequences with that of well-described plant myrosinase (*Sinapsis alba*) and insect β-glucosidases (*B. brassicae*, *Phyllotreta striolata, Spodoptera frugiperda*) shows conservation of all critical enzymatic site residues^25^,^26^, suggesting that *Psyttalia* enzymes are indeed functional (Fig. 6). Insect β-glucosidases differ from plant myrosinases in that they have two glutamates in the catalytic site instead of one glutamate and one glutamine. The possible role of *Psyttalia* venom GH1 β-glucosidase in host-parasitoid interaction is discussed below.

##### Heat shock proteins (HSPs)

Molecular chaperones belonging to the heat shock protein (HSP) class, including HSP70, calreticulin and protein disulfide isomerase (PDI), were found in variable amounts in the two species (Tables 1 and 2; Supplementary Tables S2 and S3). Endoplasmin, also identified in transcriptomic data from both species (RPKM values of 40.85 for Pl and 63.9 for Pc) was detected in Pl venom only, suggesting a lower abundance in Pc venom (Tables 1 and 2; Supplementary Tables S2 and S3). In the endoplasmic reticulum (ER), all these HSPs cooperate to form multi-chaperone complexes involved in the folding and export of secreted proteins. HSP70, PDI and endoplasmin are normally retained in the ER through the binding to specific receptors via a K/R/HDEL motif. Since these proteins are abundant in cells, we cannot totally exclude that their detection in the venom results from tissue contamination during venom collection from the gland. However, one of the venom PDIs, identified in both species, contains a substitution that changes the HDEL motif into a SDEL motif, suggesting a loss of binding and then of retention in the ER. Moreover, the binding of HSPs to ER receptors is labile and the retention is not absolute, allowing HSPs to be secreted and to play a role in intercellular transport and signaling^27^,^28^. Endoplasmin, for example, has been associated as a chaperone with the secretion of pancreatic lipases and their further internalization by intestinal cells in rat^29^.

Venom HSPs may thus play a role in stabilization of other venom proteins and/or their transport and targeting of host cells. Among these, endoplasmin is of particular interest because it is a master chaperone for TLRs, a family of LRR domain-containing proteins^30^ of which members were found in *Psyttalia* venom (see above). In accordance with a possible role of endoplasmin as a chaperone of venom TLRs, endoplasmin was only detected in the venom of Pl that contains higher amounts of LRR proteins than the Pc venom (Tables 1 and 2; Supplementary Tables S2 and S3). Interestingly these two proteins are also secreted in the venom of the braconid *A. ervi*^13^.

PDIs play a central role in the protection of disulfide bounds of secreted proteins^31^. Interestingly, the rapid expansion of a large gene family encoding PDIs specifically-expressed in the venom glands of cone snails has been recently suggested to facilitate the folding of the numerous conotoxins produced by these marine predators^32^. Here, however, phylogenetic analysis shows that *Psyttalia* venomous PDI sequences group with different PDIs from braconid wasps (Supplementary Figure S6), suggesting that no specific expansion occurred.

##### Proteins of low abundance

Proteins with similarities with serpin and enolase (Tables 1 and 2; Supplementary Tables S2 and S3) were identified. The Pl and Pc serpin sequences shared 94% identity but only the Pc sequence was complete, the Pl serpin lacking the N- and C-termini (Supplementary Figure S7). The Pc serpin contains a signal peptide as well as the consensus hinge sequence essential for the conformational change involving the RCL and necessary to inhibit the target protease (Supplementary Figure S7). An extracellular enolase was already identified in *A. ervi* injecta and also produced by specialized extra-embryonic cells (the teratocytes) and suggested to be involved in nutritional host exploitation (Table 3).

#### Proteins detected in P. lounsburyi venom only

##### Proteins of unknown function

Among the five different unisequences identified (Table 1; Supplementary Table S2), Pl_011877 and Pl_014442 have the higher RPKM values, and the predicted proteins have a low MW (less than 10 kDa). Abundant transcripts encoding low MW toxin-like peptides were previously identified in *A. ervi* venom^13^ but no toxin-like signature was found in Pl venom proteins.

##### Proteins of low abundance

All the other proteins found in *P. lounsburyi* venom only were in low abundance (Table 1; Supplementary Table S2). Among these, an arginine kinase-like protein was identified, and similarities were found with members of the esterase/lipase-like superfamily, detected in many parasitic wasp species (Table 3).

#### Proteins detected in P. concolor venom only

##### Proteins of unknown function

Two different unisequences (Pc_015919 and Pc_012023) were detected (Table 2; Supplementary Table S3) but the sequences were not complete and the presence of a signal peptide could not be assessed. Pc_012023 (rank 5 based on RPKM values) was one of the most intense 2D spots at less than 15 kDa (Fig. 4). Pc_015919 was better ranked based on RPKM values, but the corresponding spot was less intense (Fig. 4) and the number of peptide matches was lower (Table 2; Supplementary Table S3), suggesting a lower abundance in venom.

##### Annexin

One unisequence, corresponding to 16 peptide matches, had similarities with annexins (Table 2; Supplementary Table S3). This protein contains a 20 aa N-terminal signal peptide followed by two annexin domains. Based on data from their role in mammalian parasites, *P. concolor* venom annexin might be involved in binding and cell internalization of other venom proteins (Table 3).

##### Proteins of low abundance

Among these, a secreted phospholipase A2 (PLA2s) and a retinoid-inducible serine carboxypeptidase (Table 2; Supplementary Table S3) were identified (Table 3).

## Conclusions

Large scale combined “omics” studies have recently increased knowledge of the nature and diversity of the venom content of parasitoid wasps^3^,^4^. Yet, very few studies were designed to evaluate how far closely-related parasitoid species differ in venom composition. Venom glands of the Braconidae *M. hyperodae* and *M. aethiopoides* were shown to express a similar set of genes^20^ but analyses relied on less than ten genes, and proteomics data were only available for *M. hyperodae*. More recently, we evidenced striking differences in venom composition of the closely-related Figitidae *L. boulardi* and *L. heterotoma*^7^, *Drosophila* parasitoids differing in their host range. We observed here a rather similar protein composition of the venom of *P. lounsburyi* and *P. concolor*, albeit they also differ in their host specificity level. The host range is determined by both behavioral and physiological traits that include host choice and venom adequacy to the host (i.e. “virulence”). In some taxa, the diversity of venom composition could then be decoupled from the diversity of parasitized hosts *in natura* if specialization mainly relies on behavioral traits. For instance, although *P. lounsburyi* is a specialist of the olive fly in the field, it can develop in laboratory conditions on some non-natural hosts such as *Ceratitis capitata*. Similarly, although intraspecific variation in *P. lounsburyi* venom was notably evidenced for a LRR protein, the difference between *Psyttalia* strains from distant geographic origin was much lower than observed between *L. boulardi* strains^7^. Whether the level of diversity of venom differs among taxa (being lower in Braconidae than in Figitidae), or the extensive venom variation in *Leptopilina* wasps is specific of these genus/species remains an open and stimulating question.

Unfortunately, a majority of the most abundant unisequences, either common or specific to each *Psyttalia* species, had no predicted function. It is thus difficult to make assumptions about how these wasps counteract the host immune defense and regulate the host physiology, and whether they use similar mechanisms. Yet, the identification of a GH1 β-glucosidase as one of the most abundant venom protein in both species suggests a possible role of this protein in parasitoid wasp’s success. In plants, glycoside compounds and hydrolytic enzymes form a classic two-component defense system, with glycosides inducing biological effects after being activated by the enzymes. The vast array of secondary metabolites produced is used as a protection against phytophagous organisms and pathogens^33^,^34^. Hydrolysis-products can indeed be repellent or toxic to insects, nematodes, fungi and bacteria, and they also serve as volatile attractants for specialist herbivores and their parasitoids^35^.

Plant compounds and endogenous glycosidases are usually stored in separated compartments so that activation only occurs upon tissue damage. To reduce toxic effects, some phytophagous insects were shown to downregulate their gut glycosidases while others have even evolved their own glycosylation system to reglycosylate the produced aglycons. The neo-formed glycosides can then possibly be stored, similarly to the ingested plant glycosides that are indeed sequestered by several insect species. By doing so, these insects prevent the production of toxic compounds^36^,^37^,^38^, and some even use them for their own defense^39^.

The main *Psyttalia* hosts, *B. olea* and *C. capitata*, oviposit in developing olives and fruits that contain large quantities of various glycoside compounds. Olive is particularly rich in phenol-glucosides such as oleuropein, verbascoside and rutin, which accumulate during its development while *B. olea* is growing inside^40^. Oleuropein was shown to be converted into a toxic compound with a glutaraldehyde-like structure – a potent protein crosslinker – by a defense-related olive β-glucosidase^41^. Assuming that fly larvae use a sequestering mechanism to survive within fruits during development, the injection of *Psyttalia* venom β-glucosidase inside their body might result in a burst of toxic compounds that could weaken the host and increase the parasitoid probability of success. The release of sugar moieties from glycosides could also increase the amount of energy available for the developing parasitoid larvae. Although this is an attractive hypothesis, the fact remains that the alleged role of *Psyttalia* venom β-glucosidase is based on the sequestration of phenolic glycosides by the olive fly larvae, which has not been tested yet.

The importance of a tri-trophic understanding of plant-herbivore and herbivore-predator/parasitoid interactions is increasingly recognized. For instance, the role of glucosides/glucosinolates has started to be evaluated not only in insect-plant interactions but also for their cascading effects on the performance of herbivore enemies through metabolic impacts or emission of volatile products^39^. The report of the large production of a β-glucosidase in a parasitoid venom suggests that this enzyme might participate in parasitoid phenotypes such as counter-defense and parasitism success, in addition to its well-described defensive role in plants and herbivore insects.

Altogether, this study illustrates that parasitoid venom is a complex mixture of proteins whose relative abundance in a given species or group is still hardly explained. The study of a new species often reveals new types of abundant venom molecules, as exemplified here with the β-glucosidase, and it highlights the role of gene duplication in the rapid evolution of venom and acquisition of new features. Deciphering the role of major venom proteins in *Psyttalia* parasitism success, especially those with no predicted function, is a key challenge. This will require further exploration using techniques such as RNAi, demonstrated as an efficient approach for impairing the production of parasitoid venom proteins^42^.

## Material and Methods

### Biological material

The South African (SA) and Kenyan (K) strains of *P. lounsburyi* (Pl) were previously described^43^. They were reared on a laboratory strain of the fruit fly *C. capitata* under a 16:8 h light/dark cycle at 22 °C. The *P. concolor* (Pc) population was collected in 2010 in Sicily (Italy) and reared for one generation on *C. capitata* under the same conditions, prior to analysis. *C. capitata* is a natural host for *P. concolor* but it is used as a substitute host for *P. lounsburyi* due to the difficulties of rearing the main natural host, *Bactrocera oleae* (the olive fly).

### Light, fluorescence, and transmission electron microscopy

Light and fluorescence microscopies were performed using epifluorescent microscopes fitted with differential interference contrast (DIC) optics (Imager Z1, Zeiss) fitted with a black and white camera (Axiocam MRm, Zeiss). Images were pseudo-colorized digitally (Adobe Photoshop).

For transmission electron microscopy (TEM), blocks were prepared from 10 ovaries or 10 venom glands per sample. Dissected samples were pooled into 100 μl of Ringer’s saline (KCl 182 mM; NaCl 46 mM; CaCl_2_ 3 mM; Tris-HCl 10 mM) in a centrifuge vial on ice. An equivalent volume of fixative (4% glutaraldehyde (Sigma) in 0.2 M sodium cacodylate buffer, pH 7.2) was then added and the sample was kept for 24 h at 4 °C. Fixed samples were centrifuged (500 × g, 10 min) to pellet tissues and remove the fixative prior to post-fixation in 2% osmium tetroxide in cacodylate buffer. Following dehydration in graded series of ethanol solutions, samples were embedded in Epon. Sample sections were cut with a diamond knife using a LKB ultramicrotome, mounted on copper grids, stained with uranyl acetate and lead citrate, and observed with a Zeiss EM10CR electron microscope at 80 kV.

### Total RNA isolation and cDNA library construction

The transcriptomic analysis was performed from samples of 100 Pl and 100 Pc venom glands using Illumina RNA-Seq. To improve *de novo* assembly for Pl, we also generated Sanger sequences from samples of 50 venom glands, and 454 sequences from full insect bodies of 85 males and 85 females obtained from six siblings (Supplementary Fig. S1). Pl and Pc venom glands were dissected in Ringer’s saline and stored at −80 °C. Total RNA was extracted using TRIzol Reagent (Invitrogen) according to manufacturer’s instructions, and quality was checked using an Agilent BioAnalyzer. cDNA library construction for Illumina RNA-Seq and 454 sequencing was performed by Beckman Coulter Genomics (USA). cDNA library used for Sanger sequencing was constructed from 1 μg of total RNA using the Creator SMART cDNA Library Construction Kit (Clontech). Ligation products were transformed into ElectroMax DH10 B *Escherichia coli* competent cells (Invitrogen).

### Sequencing and assembly

Illumina RNA-Seq sequencing (HiSeq 2000, 2 × 75 pb), 454 sequencing (454 GS-FLX Titanium platform) and trimming were performed by Beckman Coulter Genomics. Quality of Illumina raw reads was controlled using FastQC software and reads were cleaned by removing low quality sequences and reads containing N or adaptor sequences. For Sanger sequencing, a total of 2,000 clones were analyzed by the Genoscope (CEA, Evry, France) on an ABI sequencer using the standard M13 forward primer and BigDye terminator cycle sequencing kit (Applied Biosystems, Foster City, CA, USA). Sanger ESTs were then trimmed using TIGR SeqClean software.

For each species, we performed *de novo* transcriptome assembly using Velvet/Oases assembler (https://www.ebi.ac.uk/~zerbino/oases/) after the filtering process of Illumina raw reads. The first assembly step used a multiple kmer approach with kmer size ranging from 45 to 65 (k = 45, 55, 65 and coverage = 2). A meta-assembly (kmeta = 51, coverage = 1) was then performed using all previously obtained transcripts (>100 bases long) (Supplementary Fig. S1). At both assembly steps, we used CD-HIT-EST to remove the shorter redundant transcripts entirely covered by other transcripts with more than 99% identity. Finally, a clustering of transcripts was performed using TIGR-TGICL. To improve the quality of the assembly for Pl, we included the cleaned 454 and Sanger sequences as long sequences (minimum size of 200 bases) or otherwise as short sequences, in addition to the short Illumina reads.

### Sequence annotation and analysis

To identify similarities with known proteins, the unisequences were compared to NCBI non-redundant protein sequence database, UniProtKB/Swiss-Prot database, insect predicted proteome databases (*Drosophila melanogaster* v5.46 and *Nasonia vitripennis* v1.2) and all braconid venom proteins found in UniProtKB (96 venom proteins from 9 different braconid species), using blastx with a cut-off e-value of 1e-7. Comparisons with previously published venom gland transcriptomes of *A. ervi*^13^ and *Leptopilina* spp^7^ were performed using tblastx with a cut-off e-value of 1e-7. Search for nudivirus/baculovirus-related specific genes was performed using tblastn with a cut-off e-value of 1e-1.

ORF prediction and translation were performed with FrameDP (https://iant.toulouse.inra.fr/FrameDP/), signal peptide prediction with SignalP (http://www.cbs.dtu.dk/services/SignalP/), and prediction of N-Glycosylation sites with the NetNGlyc 1.0 Server (http://www.cbs.dtu.dk/services/NetNGlyc/). Search for protein domains was achieved using PfamScan (ftp://ftp.sanger.ac.uk/pub/databases/Pfam/Tools/) and CD-Search against Conserved Domain Database (CDD) at NCBI (http://www.ncbi.nlm.nih.gov/Structure/cdd/cdd.shtml). Identification of leucine-rich repeats (LRR) in protein sequences was done using LRRfinder (http://www.lrrfinder.com/).

Search for DUF4803 domain-containing, GH1 β-glucosidase and protein disulfide isomerase sequences was performed using blastp at NCBI (http://www.ncbi.nlm.nih.gov/blast/) and HMMsearch from the HMMER package (http://hmmer.org/). Pairwise and multiple amino acid sequence alignments were respectively obtained using the Needleman-Wunsch algorithm and MAFFT implemented in Geneious software (Biomatters). Phylogenetic analyses were performed using maximum likelihood (ML) with PhyML (http://phylogeny.lirmm.fr/). ProtTest was used to select the best fit model of amino acid substitution for ML phylogeny (https://github.com/ddarriba/prottest3).

Gene functions and GO terms were automatically assigned to the predicted proteins based on the identification of domains with PfamScan. Only the root domain of the hierarchical domain organization available from EBI was conserved. Comparison of GO terms between Pc and Pl unisequences and homogenization of the annotation level were performed using GO slim terms.

### Differential expression analysis

For each species, we used bowtie (http://bowtie-bio.sourceforge.net/) to map back all input trimmed Illumina raw reads (minimum size of 30 bases) to the assembled transcriptome with up to 3 nucleotides mismatches allowed. To compare the unisequence expression levels, the number of mapped raw reads for each transcript was normalized with the RPKM (reads per kilobase per million reads), using the R package edgeR (https://bioconductor.org/packages/release/bioc/html/edgeR.html).

### SDS-polyacrylamide gel electrophoresis of venom and protein identification

The proteomic analysis was performed independently on Pc and Pl wasp venom (Supplementary Fig. S1). Venom apparatus were dissected from 50 individuals per sample and glands were collected in 25–50 μl of Ringer’s saline supplemented with a protease inhibitor cocktail (Sigma). Glands were opened to release the venom and centrifuged for 5 min at 500 × g to remove residual tissues.

For 1D SDS-PAGE, samples were mixed with 4× Laemmli buffer containing β-mercaptoethanol (v/v) and boiled for 5 min. Proteins were then separated on a 6–16% linear gradient SDS-PAGE and the gel was silver stained as previously described^7^,^13^.

For isoelectric focusing (IEF), samples were prepared by boiling the protein solution for 5 min with 4% (v:v) of a denaturing solution (0.15 M dithioerythritol, 10% SDS). After cooling, the samples were mixed with an equal volume of a solution containing 9.2 M urea, 0.1 M dithioerythritol, and 2% CHAPS. IEF was performed using slab gel. Slab gels were made on glass tubes 14 cm in length (1.5 mm internal diameter) that were filled with 4% acrylamide, 9.2 M urea, 2% ampholytes [1% pH 3–10 (Pharmacia) and 1% pH 2–11 (Servalytes)], and 2% CHAPS. Isoelectric focusing was run in two steps: a first run at 20 mA, 0.1 W/tube, 700 V for a total of 10,000 V/h, followed by a second run at 20 mA, 0.1 W/tube, 3,000 V for a total of 2,000 V/h. For the second dimension, 6–16% linear gradient SDS-PAGE was used. IEF gels were incubated with 4x Laemmli buffer containing ß-mercaptoethanol and loaded on top of the 1D SDS-PAGE. After separation, proteins were silver stained as previously described^7^,^13^.

Identification of proteins by mass spectrometry was performed on 1D bands and 2D spots excised from the gels as previously described^7^,^13^. MS/MS data analysis was performed with the Mascot software (http://www.matrixscience.com) licensed in house using the combined Pc and Pl unisequences and non-redundant NR (NCBI). Data validation criteria were (i) one peptide with individual ion score above 50 (the Mascot significant identity threshold corresponding to p <0.05 is 45 in our case) or (ii) at least two peptides of individual ion score above 20 (corresponding to 1% probability that a peptide spectrum match is a random event). The Mascot score was calculated as -10*LOG10(**P**). The calculated FDR (based on an automatic decoy database search) ranged from 0 to 1.4% depending of the individual gel analysis. Mascot analysis was performed with a fragment ion mass tolerance of 0.30 Da and a parent ion tolerance of 0.30 Da. Carbamidomethyl of cysteine was specified in Mascot as a fixed modification, and oxidation of methionine as a variable modification. The maximum missed cleavage allowed was set to 2.

## Additional Information

**Accession codes**: *P. concolor* and *P. lounsburyi* raw Illumina sequencing data are available in the GenBank Sequence Read Archive (SRA) database under the accessions SRR1593901, SRR1593902, SRR1593906 and SRR1593907, and raw 454 sequencing data for *P. lounsburyi* under the accession SRR1593908. All trimmed ESTs for *P. lounsburyi* can be found in the GenBank dbEST repository under the accessions: JZ818733 - JZ820447. The Transcriptome Shotgun Assembly (TSA) projects were deposited in GenBank under the accessions GCDX00000000 (*P. concolor*) and GCEQ00000000 (*P. lounsburyi*). Versions in this paper are the first versions GCDX01000000 and GCEQ01000000

**How to cite this article**: Mathé-Hubert, H. *et al*. Comparative venomics of *Psyttalia lounsburyi* and *P. concolor*, two olive fruit fly parasitoids: a hypothetical role for a GH1 β-glucosidase. *Sci. Rep.* **6**, 35873; doi: 10.1038/srep35873 (2016).

## Supplementary Material

Supplementary Information

Supplementary Dataset 1

Supplementary Dataset 2

## Figures and Tables

**Table 1:** Putative *P. lounsburyi* venom proteins classified based on RPKM values.

Rank	Sequence	RPKM	Mascot	Putative function	Signal peptide	Homolog in *P. concolor*		Homolog in venom of other Braconidae
						Sequence	Rank	
*1*	*Pl_004867*	*1963.84*	*71*		*Yes*	*Pc_009390*	*3*	
*2*	*Pl_009581*	*1865.04*	*11*	*Leucine-rich repeat protein*	*Yes*			*Ae*
*3*	*Pl_011877*	*1538.27*	*54*		*Yes*			
4	Pl_014442	816.50	10		?			
*5*	*Pl_010740*	*738.87*	*104*	*DUF4803 domain-containing protein*	*Yes*	*Pc_014641*	*12*	*Ci, Md, Mh*
*6*	*Pl_011340*	*691.22*	*37*	*DUF4803 domain-containing protein*	*Yes*	*Pc_010911*	*10*	*Ci, Md, Mh*
7	Pl_002959	659.87	5		Yes			
*8*	*Pl_006410*	*627.83*	*37*	*Neprilysin-like metalloprotease*	*Yes*	*Pc_006098*	*8*	*Mh*
*9*	*Pl_003816*	*574.51*	*33*	*DUF4803 domain-containing protein*	*Yes*	*Pc_013625*	*1*	*Ci, Md, Mh*
*10*	*Pl_006199*	*476.04*	*153*	*DUF4803 domain-containing protein*	*Yes*	*Pc_014641*	*12*	*Ci, Md, Mh*
*11*	*Pl_002819*	*272.37*	*93*	*GH1 β-glucosidase*	*?*	*Pc_001157*	*7*	*Ae*^b^*, Md*^c^
12	Pl_010491	263.42	1	Calreticulin	?	Pc_015292	17	Mh
*13*	*Pl_002333*	*242.50*	*90*		*Yes*	*Pc_006379*	*4*	
14	Pl_014829	186.32	8	Reprolysin-like metalloprotease	?			Ci, Md
15	Pl_002212	143.39	3		Yes			
16	Pl_006057	135.90	1	Esterase/lipase-like	?			Ci, Md
17	Pl_013024	115.21	10	Neprilysin-like metalloprotease	?	Pc_006098	8	Mh
18	Pl_014435	112.79	0^d^	Protein disulfide isomerase	Yes	Pc_014697	15	Ae
*19*	*Pl_014734*	*85.45*	*24*	*Heat shock protein 70*	*?*	*Pc_008008*	*18*	*Ae*^b^
20	Pl_002507	63.77	3	Protein disulfide isomerase	Yes			Ae^b^
21	Pl_008373	40.85	8	Endoplasmin	Yes			Ae
22	Pl_003563	35.95	6	DUF4803 domain-containing protein	Yes	Pc_002889		Ci, Md, Mh
23	Pl_011829	22.74	16	Protein disulfide isomerase	Yes	Pc_010489	19	Ae^b^
24	Pl_001931	21.08	6	Puromycin-sensitive aminopeptidase^a^	?			
25	Pl_007984	17.02	2	Enolase	?	Pc_009146	21	
26	Pl_011015	6.13	5	Arginine kinase-like protein	?			
27	Pl_010999	2.56	13		?			
28	Pl_009261	2.43	4	Esterase/lipase-like	?			
29	Pl_000063	2.28	14	Serpin	?	Pc_007867	24	Ae, Md
30	Pl_012461	2.02	6	Leucine rich repeat protein	?			Ae
31	Pl_013792	1.47	2	Neprilysin-like	Yes			Ae, Mh
32	Pl_004270	1.31	5	Glycogen phosphorylase^a^	?			

Ae, *Aphidius ervi*^13^. Ci, *Chelonus inanitus*^11^. Md, *Microplitis demolitor*^12^. Mh, *Microctonus hyperodae*^20^. Abundant proteins (RPKM > 50 and Mascot matches > 10) are in italics.

^a^Unisequences for which secretion could not be predicted and that are typical cellular proteins.

^b^Proteins identified in the analysis of *A. ervi* venom apparatus but not considered as venom proteins due to a highly conservative approach.

^c^See Burke & Strand^12^ (Supplementary Table 2, locus comp21422_c0).

^d^Protein not found in the proteomic analysis but with RPKM > 50 and for which a homolog was found in *P. concolor*.

**Table 2:** Putative *P. concolor* venom proteins classified based on RPKM values.

Rank	Sequence	RPKM	Mascot	Putative function	Signal peptide	Homolog in *P. lounsburyi*		Homolog in venom of other Braconidae
						Sequence	Rank	
*1*	*Pc_013625*	*3110.46*	*59*	*DUF4803 domain-containing protein*	*?*	*Pl_003816*	*9*	*Ci, Md, Mh*
*2*	*Pc_015919*	*1626.40*	*12*		*?*			
*3*	*Pc_009390*	*1583.31*	*210*		*Yes*	*Pl_004867*	*1*	
4	Pc_006379	1507.73	8		?	Pl_002333	13	
*5*	*Pc_012023*	*1428.59*	*34*		*?*			
6	Pc_012375	1326.30	8	Reprolysin-like metalloprotease	?			Ci, Md
*7*	*Pc_001157*	*1196.52*	*84*	*GH1 β-glucosidase*	*Yes*	*Pl_002819*	*11*	*Ae*^b^, *Md*^c^
*8*	*Pc_006098*	*1178.46*	*47*	*Neprilysin-like metalloprotease*	*?*	*Pl_013024*	*17*	*Mh*
9	Pc_014667	1152.41	9	DUF4803 domain-containing protein	Yes			Ci, Md, Mh
*10*	*Pc_010911*	*947.92*	*71*	*DUF4803 domain-containing protein*	*Yes*	*Pl_011340*	*6*	*Ci, Md, Mh*
11	Pc_002246	580.77	3	Phospholipase A2	?			Md
*12*	*Pc_014641*	*441.52*	*57*	*DUF4803 domain-containing protein*	*Yes*	*Pl_010740*	*10*	*Ci, Md, Mh*
*13*	*Pc_007330*	*360.78*	*16*	*Annexin*	*Yes*			
14	Pc_009900	346.13	5	Serine carboxypeptidase	Yes			Md
*15*	*Pc_014697*	*259.63*	*32*	*Protein disulfide isomerase*	*Yes*			*Ae*^b^
16	Pc_002889	243.19	2	DUF4803 domain-containing protein	Yes	Pl_003563		Ci, Md, Mh
17	Pc_015292	236.32	3	Calreticulin	Yes	Pl_010491	12	Mh
*18*	*Pc_008008*	*227.98*	*43*	*Heat shock protein 70*	*Yes*	*Pl_014734*	*18*	*Ae*^b^
*19*	*Pc_010489*	*199.42*	*13*	*Protein disulfide isomerase*	*Yes*	*Pl_011829*	*22*	*Ae*^b^
20	Pc_009911	102.94	1	Leucine-rich repeat protein	Yes			Ae
21	Pc_007769	60.85	0^d^	Protein disulfide isomerase				
22	Pc_009146	53.51	9	Enolase	?	Pl_007984	24	
23	Pc_015675	39.64	3	Leucine-rich repeat protein	Yes			Ae
24	Pc_002924	36.88	3	Ezrin/radixin/moesin family^a^	?			
25	Pc_007867	32.85	12	Serpin	Yes	Pl_000063	28	Ae, Md
26	Pc_000616	29.37	5	Neprilysin-like metalloprotease	?			Mh
27	Pc_016110	12.69	4	Aldehyde dehydrogenase^a^	?			
28	Pc_005686	5.03	1	Leucine-rich repeat protein	?			Ae
29	Pc_007684	4.49	1	Leucine-rich repeat protein	?			Ae
30	Pc_009846	2.31	3	Adenosylhomocysteinase^a^	?			

Ae, *Aphidius ervi*^13^. Ci, *Chelonus inanitus*^11^. Md, *Microplitis demolito*^12^*r*. Mh, *Microctonus hyperodae*^20^. Abundant proteins (RPKM > 50 and Mascot matches > 10) are in italics.

^a^Unisequences for which secretion could not be predicted and that are typical cellular proteins.

^b^Proteins identified in the analysis of *A. ervi* venom apparatus but not considered as venom proteins due to a highly conservative approach.

^c^See Burke & Strand^12^ (Supplementary Table 2, locus comp21422_c0).

Protein not found in the proteomic analysis but with RPKM > 50 and for which a homolog was found in *P. lounsburyi*.

**Table 3:** *Psyttalia* venom proteins with a putative function: Biochemical function, occurrence in venom of parasitoids and previously demonstrated or proposed role in parasitism.

Protein function	General properties and comments
Annexin	Annexins are a family of Ca2^+^-dependent lipid binding proteins believed to be engaged in membrane transport processes, although recent work suggests a more complex set of functions. Annexins normally lack signal sequences for secretion, but some members of the family have been identified extracellularly where they can act as receptors^44^. Annexins had never been described so far in the venom of parasitoids. However, some data suggest that different mammalian parasite clades possess annexins with unique properties that can be secreted and are likely involved in host-parasite interactions and host immune-modulation^45^. Moreover, some parasitic nematodes secrete an annexin-like effector into host root cells that may mimic plant annexin function during the parasitic interaction^46^. At last, it has been shown that annexins are also involved in the binding and internalization of toxins in eukaryotic cells^47^.
Arginine kinase	Arginine kinase plays a crucial role in the energy metabolism of insects and other invertebrates through the use of ATP to catalyze the phosphorylation of arginine in phosphoarginine. This enzyme was detected in the venom of *Pteromalus puparum*^48^ and *Leptomastix dactylopii*^49^, but its role in the host-parasitoid interaction is unknown.
Calreticulin	Calreticulin is a calcium (Ca2^+^)-binding protein with multifunctional properties including chaperone functions^50^. Calreticulin was shown to inhibit host cell encapsulation in *Cotesia rubecula*^51^ and *P. puparum*^52^, although the mechanism is still unclear. Calreticulin was found in the venom of several phylogenetically distant species^4^ and seems thus largely shared among parasitoids.
Endoplasmin	Endoplasmin (alternative names: HSP90B1, GP96, GRP-94), which belongs to the heat shock protein 90 family, is a molecular chaperone located in the ER and involved in the final processing and export of secreted proteins^53^. Among parasitoids, endoplasmin has only been detected so far in the venom gland of *Aphidius ervi*^13^. This venom protein was suggested to play a role in the secretion, stabilization, transport and host cell targeting of the different *A. ervi* venom proteins.
Enolase	Enolase is a key enzyme in cell metabolism which is also associated with virulence of several pathogens^54^. An extracellular enolase was recently described in the oviposition injecta from *A. ervi*^55^ and the venom of *Toxoneuron nigriceps*^56^. Enolase is also released by teratocytes surrounding the *A. ervi* embryo^57^. This enzyme was proposed to play an important role in the regulation of the host physiology and the host nutritional exploitation^57^,^58^.
Esterase/lipase-like	Esterases and lipases belong to a superfamily of hydrolytic enzymes that act on carboxylic esters^59^. Proteins belonging to this functional class were previously found in the venom of several phylogenetically distant species^3^,^4^ and appear to be common in parasitoids. The functions of these hydrolase enzymes in host-parasitoid interactions have not been investigated yet.
GH1 β-glucosidases	GH1 β-glucosidases are a family of enzymes found from bacteria to mammals that hydrolyze glycosidic bonds from glycosides and oligosaccharides, and remove non reducing terminal glucosyl residues^60^. Among parasitoids, a β-glucosidase enzymatic activity was detected in the venom of *Pimpla hypochondriaca*^61^. A member of this enzyme family was also recently identified, but not abundant, in the venom of *Microplitis demolitor*^12^ and, in a low quantity, in a transcriptomic study of the *A. ervi* venom apparatus^13^. GH1 β-glucosidases include myrosinases that play a central role in the glucosinolate-myrosinase system, one of the best-studied activated plant defense system^33^. Some insects have co-opted this system to defend themselves against enemies, by sequestering plant-derived glucosinolates and producing their own myrosinase-like enzyme^36^,^37^,^38^.
Heat shock protein 70	Heat shock proteins 70 (Hsp70; alternative name: GRP-78) are a family of chaperones with distinct sub-cellular localization and function^62^. An Hsp70 protein was identified in the venom of the parasitoid *P. puparum*^48^ whose function remains to be elucidated.
Leucine-rich repeat protein	Leucine-rich repeats (LRRs) are motifs involved in protein-protein interactions^63^. LRRs are generally composed of 20-29 amino acid stretches rich in leucine. To our knowledge, LRR domain-containing proteins were only identified in the venom of *A. ervi* until now^13^. They were suggested to act as scavengers for the pea aphid Toll-like receptors, thus impairing the host immune response via the Toll pathway.
Neprilysin-like metalloprotease	Neprilysin-like (NEP) proteins are zinc-dependent metalloproteases belonging to the M13 peptidase family. They are involved in the degradation of a number of regulatory peptides in the nervous or immune system of mammals^64^ and insects^65^. Although they are typically membrane-bound, ectopeptidases such as NEP may also be shed from the membrane through a proteolytic process and found in the surrounding fluid^66^. NEP-like proteins were detected in the venom of the Braconidae *A. ervi*^13^, *Microctonus hyperodae*^20^, *M. demolitor*^12^ and *T. nigriceps*^56^, as well as of the Figitidae *Leptopilina boulardi*^7^. They were also found associated with the VLPs produced in the ovary of *Venturia canescens*^67^. NEP-like proteins have been hypothesized to modulate the host immune system by degrading immune-specific peptides^67^.
Phospholipase A2	Secreted phospholipases A2 (PLA2s) are a family of relatively stable enzymes found in venoms. PLA2 has *in vitro* and *in vivo* immunomodulatory effects in bee venom^68^, and neurotoxic and myotoxic effects in snake venoms^69^. This enzyme was recently detected in the venom of *M. demolitor*^12^ and *T. nigriceps*^56^, but its function in the host-parasitoid interaction is unknown.
Protein disulfide isomerase	Protein disulfide isomerases (PDIs) are enzymes involved in the folding and stabilizing of nascent polypeptides in the endoplasmic reticulum (ER) through catalysis of disulfide bond formation and isomerization^70^. Although this protein is normally recycled back to the ER from the Golgi via its C-terminal KDEL motif, secreted PDIs can escape this turnover mechanism^71^. Among parasitoids, PDIs have only been detected so far in the venom gland of *A. ervi*^13^. They have a broad substrate specificity and are involved in the folding of toxin peptides in different venomous organisms^72^,^73^.
Reprolysin-like metalloprotease	Reprolysin-like (REP) proteins are zinc-dependent metalloproteases belonging to the M12 peptidase family, commonly found as constituents of snake venom. They were previously detected in the venom of the parasitoids *Pimpla hypochondriaca*^22^, *Eulophus pennicornis*^23^, *Chelonus inanitus*^11^, *M. demolitor*^12^ and *T. nigriceps*^56^. A recombinant *E. pennicornis* venom REP-like protein was demonstrated to display toxicity toward the host and to manipulate host development^23^.
Serine carboxypeptidase	Classical serine carboxypeptidases are enzymes that hydrolyze a peptide bond at the C-terminal end of peptides and proteins. A related enzyme (Scpep1) that do not show proteolytic activity but is involved in other functions was described in mice^74^. To our knowledge, serine carboxypeptidase has only been identified so far in the venom of *M. demolitor*^12^ and *T. nigriceps*^56^. Interestingly, serine carboxypeptidases have also been described as candidate virulence factors in several pathogens^75^.
Serpin	Serpins (serine protease inhibitors) are a large family of functionally diverse protease inhibitors. They share a conserved structural architecture with an exposed reactive center loop (RCL) of about 20 amino acids, which acts as bait for target serine proteases^76^. The *L. boulardi* venom serpin LbSPNy was previously shown to be involved in host immune suppression^77^: it interferes with melanization in the *Drosophila* host through inhibition of the phenol oxidase activation. More recently, serpins were described in the venom of *A. ervi*^13^ and *M. demolitor*^12^ but their role in parasitism success is unknown.
