# Supplementary material for: Comparative venomics of Psyttalia lounsburyi and P. concolor, two olive fruit fly parasitoids: a hypothetical role for a GH1 β-glucosidase
Source: Sci Rep. 2016 Oct 25;6:35873. doi: 10.1038/srep35873 (PMC5078806; doi:10.1038/srep35873)
Supplement: Supplementary Information [file srep35873-s1.pdf]

Comparative venomics of *Psyttalia lounsburyi* and *P. concolor*, two olive fruit fly parasitoids: a hypothetical role for a GH1 β-glucosidase

Mathé-Hubert Hugo[1*], Colinet Dominique[1*], Deleury Emeline[1], Belghazi Maya[2], Ravallec Marc[3], Poulain Julie[4], Dossat Carole[4], Poirié Marylène[1$], Gatti Jean-Luc[1$]

[1] *Université Côte d'Azur, INRA, CNRS, ISA, France*

[2] *CNRS, Aix-Marseille Université, UMR 7286, CRN2M, Centre d'Analyses Protéomiques de Marseille (CAPM), Faculté de Médecine, Marseille, France*

[3] *INRA, Univ. Montpellier, UMR 1333 « Microorganism & Insect Diversity, Genomes & Interactions » (DGIMI), CC101, Montpellier Cedex 34095, France*

[4] *Commissariat à l'Energie Atomique (CEA), Institut de Génomique (IG), Génoscope, 91000, Evry, France*

[*]co-first authors, [$]co-last authors

Supplementary Table S1. General features of the *P. lounsburyi* and *P. concolor* transcriptomes and results of similarity searches.

	P. lounsburyi	***P. concolor***
DATA		
Number of trimmed paired-end reads	88,793,008	41,603,068
Number of trimmed single-end reads	2,412,351	4,498,697
Number of trimmed 454	359,589	/
Number of trimmed Sanger	1,963	/
ASSEMBLY		
**Number of unisequences** (min size 100 bp)	**16,943**	**16,360**
Min sequence length	104	103
Average sequence length	1,663	983
Max sequence length	20,780	17,046
N50 length	2,698	1,784

LIBRARY COMPARISONS		
P. lounsburyi	-	13,935
P. concolor	12,160	-

SIMILARITY SEARCHES		
With public databases		
NCBI NR	11,448	9,826
Swiss-Prot	9,162	7,365
With insect proteomes		
Nasonia vitripennis	10,830	9,134
Drosophila melanogaster	9,269	7,550
With parasitoid venom gland transcriptomes		
Aphidius ervi	1,779	1,187
Leptopilina boulardi ISm	1,616	1,043
Leptopilina boulardi ISy	1,574	1,101
Leptopilina heterotoma	2,115	1,398

TRANSLATION AND SECRETION		
Unisequences with ORF prediction	13,321	11,587
Predicted full length unisequences	3,130	1,990
Unisequences with signal peptide	905	744

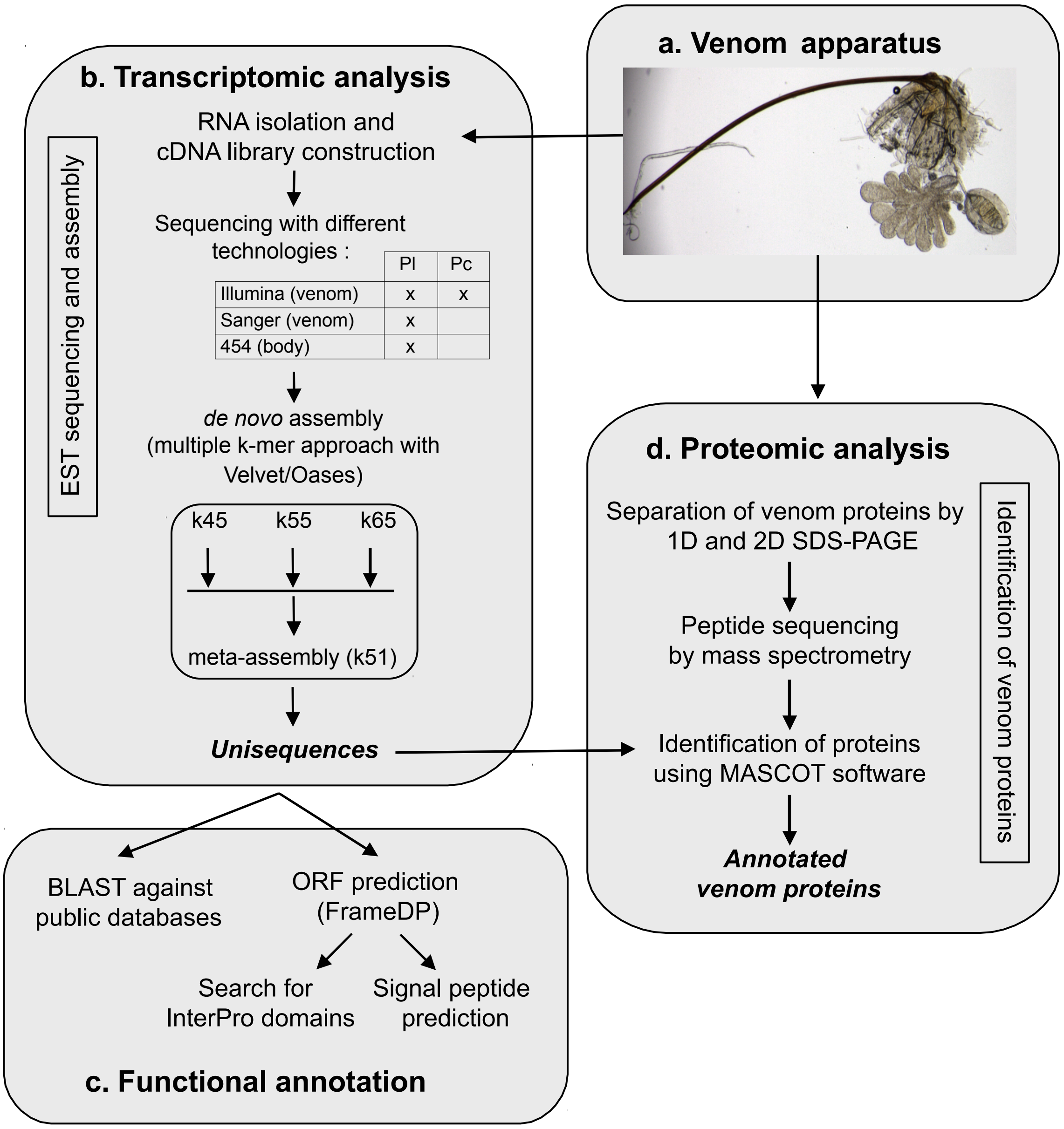

Supplementary Figure S1. Schematic representation of the combined large-scale transcriptomic and proteomic approach. Picture, venom apparatus of *P. lounsburyi*.

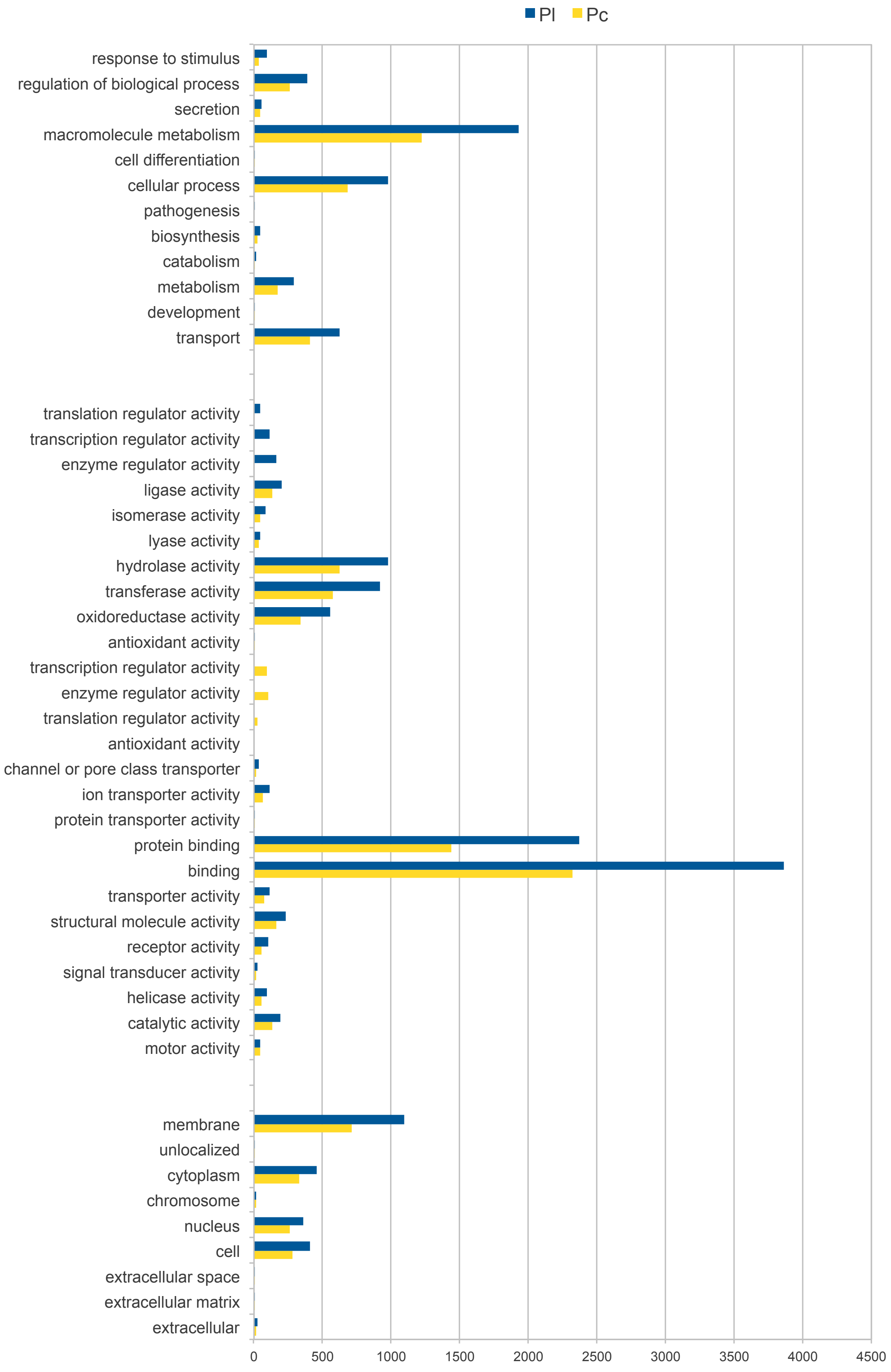

Supplementary Figure S2. Distribution of the number of unisequences associated with GO terms for Pl and Pc venom gland transcriptomes.

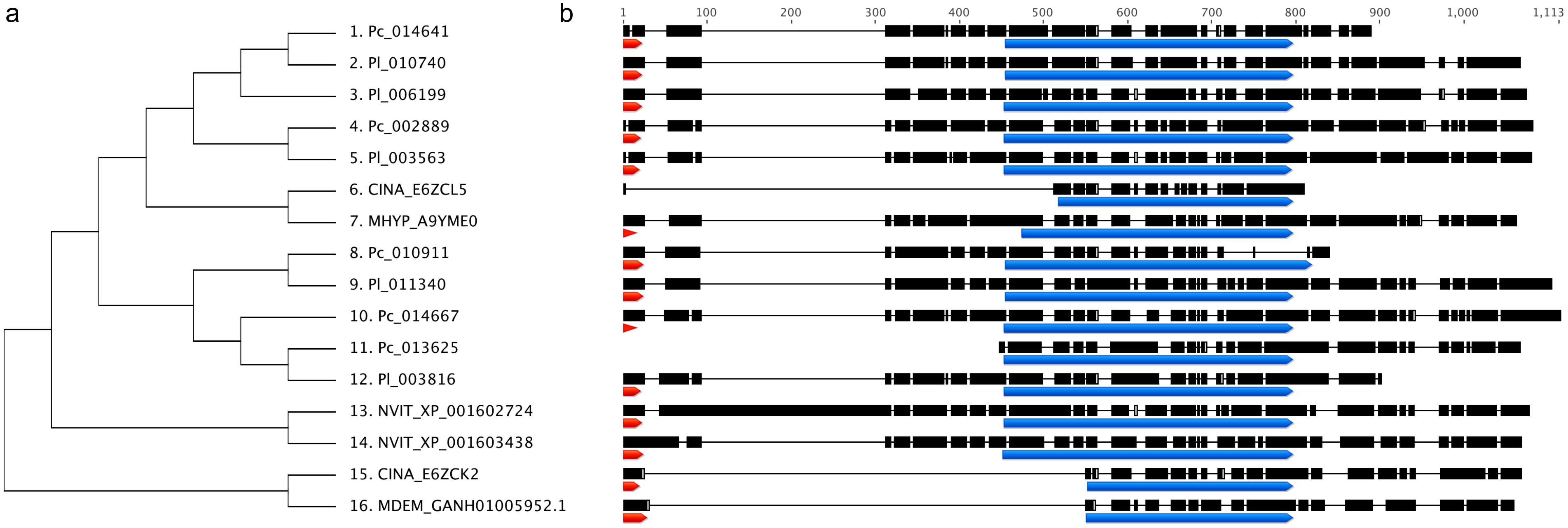

Supplementary Figure S3. a, Cladogram of venomous DUF4803 domain-containing sequences. b, Graphical view of the multiple alignment. *P. lounsburyi* (Pl_) and *P. concolor* (Pc_) sequences were aligned with *M. hyperodae* (A9YME0), *M. demolitor* (GANH01005952.1, *C. inanitus* (E6ZCL5 and E6ZCK2) and *N. vitripennis* (XP_001602724 and XP_001603438) venom protein sequences. The signal peptide and the DUF4803 domain are indicated by red and blue arrows, respectively.

```
Pc_001157  MKILNVAALLTFVPVILANDDEYLKLPPGLSIGAASAAHQIEGAWNVSDKGLNVWDEFTH
Pl_002819  ---------------ILANDDKYLKLPPGLSIGAASAAHQIEGAWNVSDKGLNVWDEFTH

Pc_001157  RKPSMVDNQTNADITSNSYYKYKEDIQIMKDIGLTHYRFSLSWARILPTGFPDKISQDGV
Pl_002819  RKPSMVENQTNADITSNSYYKYKEDIQIMKDIGLTHYRFSLSWARILPTGFPDKISQDGV

Pc_001157  QYYKNIIDELKSKGIEPFVTIYHWDHPALMGAMGSWTNELMVEWFGHYARVVFREFGPKV
Pl_002819  QYYKNIIDEVKSKGIEPFVTLYHWDHPALMGAMGSWTNELMVEWFAHYARVVFRELGPKV

Pc_001157  KFWATVNEPEMYCKLTHGRNTYAPGLNSPLFGEYICLHNLLKAHARAYQIYNDEFRADQG
Pl_002819  KFWATVNEPEMYCKLSHGRNTYAPGINSPLFGEYICLHNLLKAHARAYQIYNDEFRADQG

Pc_001157  GKIGLVPLCFHHLPASEDFTEAAEIAHQFDCGRTIHPIFSKEGDYPPIVKQRIRENSELE
Pl_002819  GKVGLVPLCFHHLPASEDFTEAAEIAHQFDCGRTIHPIFSKDGDYPPIVKQRIRENSELE

Pc_001157  GLPFSRLPEFSPYWINLLRGSADYFGLNHYSTYLVEPVPWSNKTGWYNDSGVRSFTDKNW
Pl_002819  GLPFSRLPEFSPYWINLLRGSADYFGLNHYSTYLVEPVPWSNKTGWYSDSGVRSFTDKNW

Pc_001157  AQSPAGWLKIVPEGFGSLLRLIKDKYGNPPVHVLENGIATYKGHEDYLRINYLHDYMKEM
Pl_002819  AESPAGWLKIVPEGFGSLLRLIKEKYGNPPVHVLENGIATYIGHEDYLRINYLHDYMKEM

Pc_001157  LVAINRDGCNVEVYTVWSLLDSFEWSRGYEHRFGLVEVDFDSPNRTRTPRLSTKWLREIL
Pl_002819  LVAINRDGCNVEVYTVWSLLDSFEWSRGYEHRFGLVEVDFDSPNRTRTPRLSTKWLREML

Pc_001157  AKRKLEPLKTTEFYKKSVVADTSDQGR
Pl_002819  AKRKLEPLKTTEFYKESVVADTSDQVR
```

Supplementary Figure S4. Pairwise sequence alignment of *P. concolor* Pc_001157 and *P. lounsburyi* Pl_002819 GH1 β-glucosidase sequences. Identical and similar residues are highlighted in black and grey, respectively. The predicted signal peptide in *P. concolor* sequence is indicated by a red double-sided arrow. Predicted N-glycosylation sites are printed in white on a blue background.

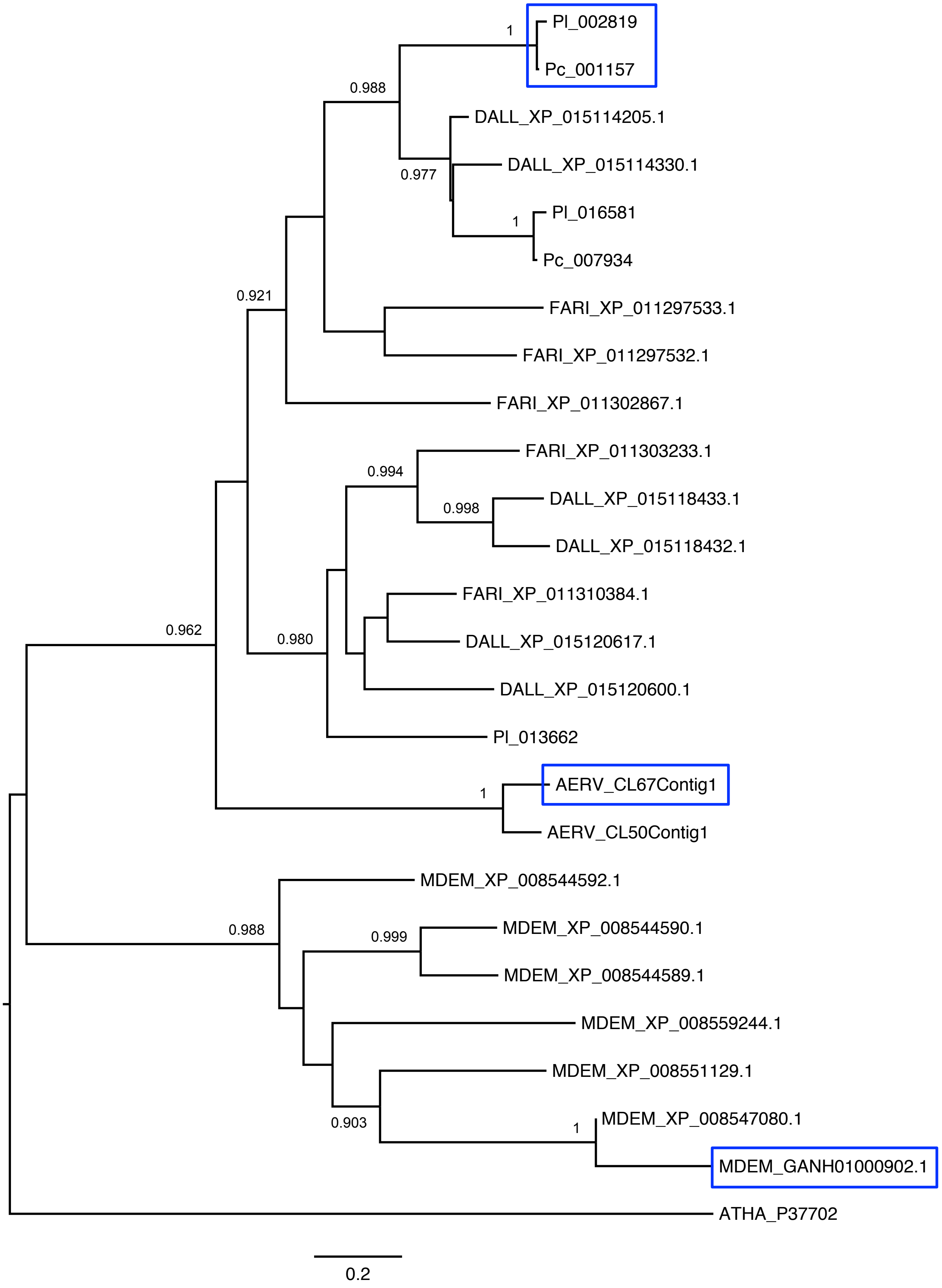

Supplementary Figure S5. Maximum-likelihood phylogenetic tree of braconid GH1 β-glucosidase sequences. Venomous or presumed venomous proteins are marked with blue rectangles. Numbers at corresponding nodes are aLRT SH-like branch support values. Only aLRT support values greater than 0.9 are shown. The outgroup is the *Arabidopsis thaliana* Myrosinase 1 sequence [Swiss-Prot: P37702]. AERV, *Aphidius ervi*; ATHA, *A. thaliana* ; DALL, *Diachasma alloeum* ; FARI, *Fopius arisanus* ; MDEM, *Microplitis demolitor*; Pc, *Psyttalia concolor* ; Pl, *Psyttalia lounsburyi*.

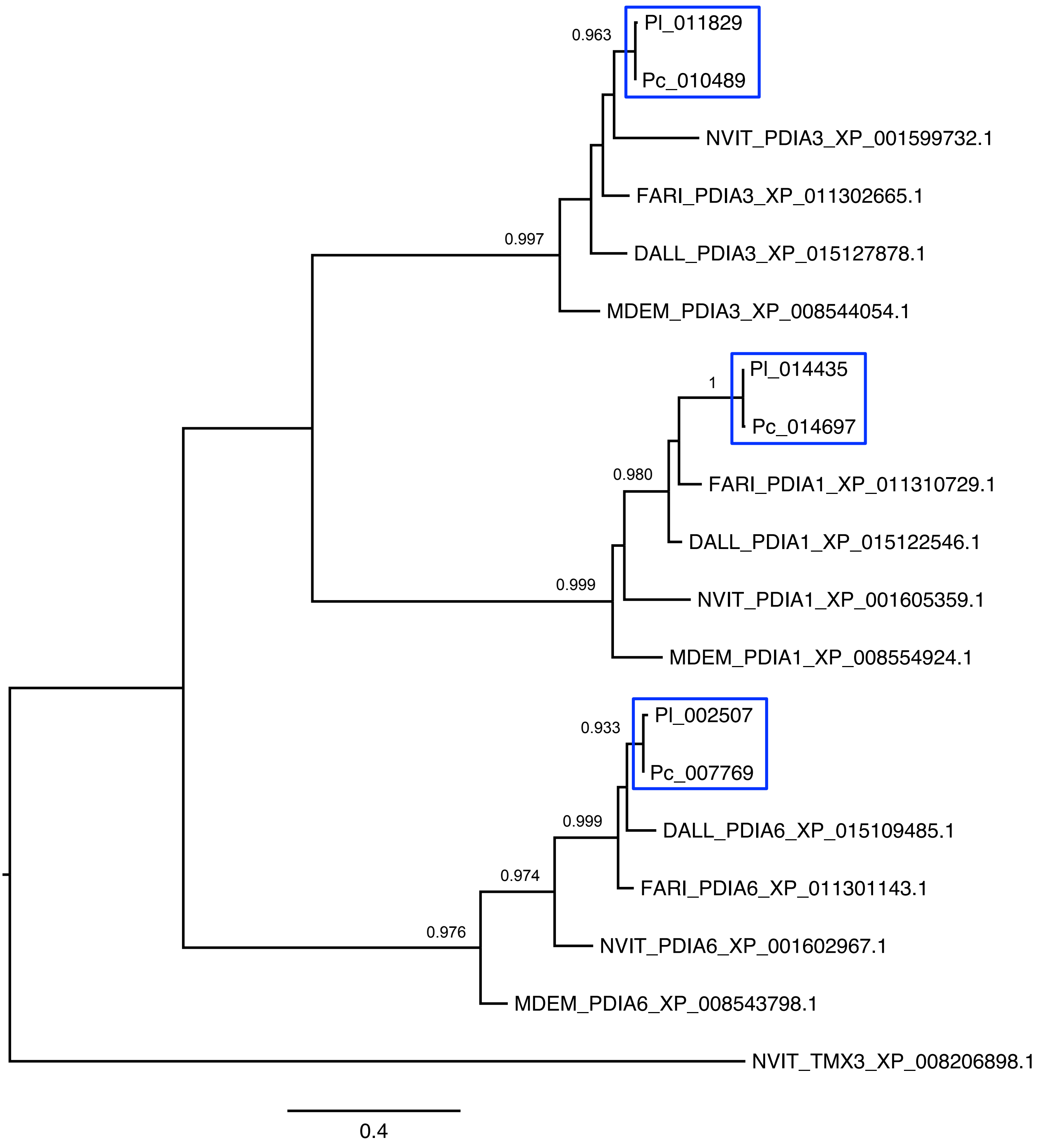

Supplementary Figure S6. Maximum-likelihood phylogenetic tree of braconidae protein disulfide isomerase (PDI) sequences. Venomous proteins are marked with blue rectangles. Numbers at corresponding nodes are aLRT SH-like branch support values. Only aLRT support values greater than 0.9 are shown. The outgroup is the *Nasonia vitripennis* PDI TMX3 sequence [GenPept: XP_008206898.1]. DALL, *Diachasma alloeum* ; FARI, *Fopius arisanus* ; MDEM, *Microplitis demolitor* ; NVIT, *N. vitripennis* ; Pc, *Psyttalia concolor* ; Pl, *Psyttalia lounsburyi*.

```
Pc_007867           MRVFLILGFAFLSSSWAMT-------------VPENPEALRMVTRGMNLFAKKFYTKSVK
Pl_000063           ------------------------------------------------------------
Ae_CL18Contig1      MNFKFGVLGLVAVSLLLASGNAKKHSSKRHLDVWLTNKARSFLMTTVNTFTGKLVEAAFN
Ae_aar0aka8ya02cm1  ------------------------------------------------------------
Md_comp17591_c0     --MIIKCLSIVIIVTISVTNSSIYDDDINMNGIIPAKPDLQFDPSAINKFSKRFYSVLAE
Hd-Ven390           ----MRFLLLISIAVWCVS------SSEAMAVQPAQSEGFQAVTKSTNAFSPNFYKHVAA
LbSPNy              -MMFTHLLSFFLIGTCSIYAVIAYGNVNYFSEELVQPLDFH---QALEKFNNDLYKNAAS

Pc_007867           DKD-DNFVMSPAGISIVTSMASFGAGGTTKTQIQTALNLP-TDDHTARSGIKSMMDQGRG
Pl_000063           -----------------------------------------------------MMDQGRG
Ae_CL18Contig1      KTQ-DSYTICPIIVWSMLTKISDLSGGQTKEQLEDFLNW--KNTSWHRLGLASLYRRYHH
Ae_aar0aka8ya02cm1  ------------------------------------------------------------
Md_comp17591_c0     YES-DNLICSPLSVYVTLFMASYGACGNTKKQLLSALSSP-NDKTITEREIQHLLYSLNN
Hd-Ven390           DTK-GNLICSPLSASMVLSMVAYGARGNTAKQMRSVLALP-EKDELAKSGFQAFVDSFKN
LbSPNy              NGSIQNVLLSPLSINILLAILAVGAAGRTRSEIVTGINQPLQSGAQILNNYKLMVENLMN

Pc_007867           LQSIELKMANKIFTTTGVEMKPEFKEITKKTFNSEAQSMDFTKP-EATETINAWAAEKTN
Pl_000063           LQSIELKMAKKIFTTTGVEMKPEFKEITKKTFNSEAQSMDFTKP-EATETINAWGCREDE
Ae_CL18Contig1      FKNMEVNSTGLIVIDKSVDLTDKNSDHLREI---KIVNVDFSKTDEAYNIINKRAEENSD
Ae_aar0aka8ya02cm1  ------------------------------------------------------------
Md_comp17591_c0     MQGAEVKLVNKIFATNKFKLQPKFTKITKEYFGSEVKRVDFINVEKTVRTINNLCANESN
Hd-Ven390           YKQVDLRLANKVFLNEGVKPKAEFSAMTKEGFRSEAQNVNFVKSAEAAKTINDWCEAQTN
LbSPNy              VTDVELQMSNAIFVDHSIRLKKSFQDELFNYFKAHEFSVNFKTPIPTVDKINGKISEQTN

Pc_007867           NKIQNLLQPDDIK--DASMVLANAVYFKGKWMKPFNAQMTMPKKFHMDDQTSKDVPMMSK
Pl_000063           HKIQNLLQPDDIK--DASMVLANAVYFKGKWMKPFNAQMTMPKKFHMDDQTSKDVPMMSK
Ae_CL18Contig1      HLINEIIPKGKFEK-DYRMVIANGGFIRGIWAVPFYPE-TSKTPFQLLDGTKKEVDMMRA
Ae_aar0aka8ya02cm1  ------------------------------------------------------------
Md_comp17591_c0     NHITDVVEPSDIE--GAEMILVSAIYFKGKWAEKFKFKWTNPYPFEIDEKTTKDVPMMFK
Hd-Ven390           NRIKDLIKPDDLDA-STAMVLVNAVYFKGNWSDKFDPANTKPRPFNVDATTKKDVPTMFR
LbSPNy              NKINNMLSTKDIDERSTKVVITNAIYFKGEW--KYKFANVTNLVFHDYHGQTKIVPTMTK

Pc_007867           MDNMFYAEL-PDMQAKIVELPYEREKDSPNKYVSMFIIVPNEVNGLKDIEKNMETINMDM
Pl_000063           MDNMFYAEF-PDMQAKIVELPYEREKDSPNKYVSMFIIVPNEVNGLKDIEKNMETINLDM
Ae_CL18Contig1      RGIFPIGYLNPDYSAKIIQLDY--LWDNYNTAVNNYLVIPEEDVGFDKVEKLLYNFDFSN
Ae_aar0aka8ya02cm1  -----------------------------------FIIVPFQIEGLKHIEDNIGKLNLTN
Md_comp17591_c0     EAKFYWGYI-RAVKSRFIKLPY--ESHGKKETIEMIIILPHNGVNIHDVENNINKIDFTR
Hd-Ven390           SGSYNFGVL-DDLKAKFVEIPY--KSEGPNDAMSMFVIVPDAVDGLAELEKNIEKVSMDR
LbSPNy              TGPYRVSFGDTAIKARMIELPY--KGD----ELSMLIVIPYEMNGLDDVESSLERVNLRN

Pc_007867           VRKHGSMRDVILDMPKFKMESTNNLRPVMEEMGMTDMFSDKADFTGITDSPPLKISKAMQ
Pl_000063           VRKHGSMRDVILDMPSLK-------------------WS---------------------
Ae_CL18Contig1      FE-VDEVANINLLMPKFKLETSVELKKPLNDIGVTKIWSKESDLSGFSKN--TYVNEFFH
Ae_aar0aka8ya02cm1  FVHNKYTSLIRLYLPKFKLETKIDLKRHLKKMGINRAFRTNANFKGISGNQNVFINKVVQ
Md_comp17591_c0     LK--GSTMKMALHLPKFKIESKFNLKPTLQDVGITQMFKDTADFSGIIKNTKLKVSKIIQ
Hd-Ven390           LL-NSRKQDVNLYLPKFKIESDIPLNAVLAKMGMTDMFSDAADFTGIIDSPRLAVSKVVQ
LbSPNy              HIKSLMPFEVKLNLPKFRVEATTDLYDALNKMGINEAFTDIANFSRITDG-NLSVSKMMH

Pc_007867           KAMIDVNEEGSE---AAAVTTMIAVPMSMPMPQELPVTLTIDRPFYFSIVIVNNELEGGQ
Pl_000063           -----------------------------------PPKI---------------------
Ae_CL18Contig1      KTGIVVDPFFNE---KTKSYIA---------PKGPVTNVVVDKPFFWITVSDHG------
Ae_aar0aka8ya02cm1  KATIYVNKDGTDDDHEAKIYGVDHILLKY-------MTIKVDHPFLFVIATNNN------
Md_comp17591_c0     KAFIEVNEEGTE---AAAVTGM-----KMK-SRSSPLQFTVNRPFLCVIVKSNS------
Hd-Ven390           KAFIEVNEEGSE---AAAATDVGIMLLSIR-PTYPVINFNVDRPAHFIIATKSN-----D
LbSPNy              KTLIEVNENGAE---AAAASVA---VLGFRSMKSDPPEFNANHPFHYKIIKSIDK----N

Pc_007867           ARTMLFTGRVMDPTSN*
Pl_000063           -----------------
Ae_CL18Contig1      --VVILFSRITNPAYQ-
Ae_aar0aka8ya02cm1  ---ILFTGRVTNPLL--
Md_comp17591_c0     -NTPLFYARIMDPTAN-
Hd-Ven390           QNAILFRGSVHDPLAN-
LbSPNy              NHVVLFAGNVKHIQ---
```

Supplementary Figure S7. Multiple alignment of serpin sequences. *P. concolor* (Pc_007867) and *P. lounsburyi* (Pl_000063) serpins were aligned with *A. ervi* (JAC59136 and JAC59130), *M. demolitor* (GANH01000239), *H. didymator* (Burke and Strand, 2014) and *L. boulardi* LbSPNy (ACQ83466) venom serpins. Identical and similar residues are highlighted in black and grey, respectively. The signal peptide and the hinge region found in P. concolor serpin region are indicated by red and blue double-sided arrows respectively.
